# PIEZO channels as multimodal mechanotransducers

**DOI:** 10.1042/BST20240419

**Published:** 2025-02-12

**Authors:** Jérôme J. Lacroix, Tharaka D. Wijerathne

**Affiliations:** Department of Biomedical Sciences, Western University of Health Sciences, Pomona, CA 91766, U.S.A

**Keywords:** Mechanosensitive ion channels, PIEZO1, PIEZO2, Mechanotransduction, Fluid shear sensing, Curvature-based gating, Force-from-lipids, Force-from-filaments

## Abstract

All living beings experience a wide range of endogenous and exogenous mechanical forces. The ability to detect these forces and rapidly convert them into specific biological signals is essential to a wide range of physiological processes. In vertebrates, these fundamental tasks are predominantly achieved by two related mechanosensitive ion channels called PIEZO1 and PIEZO2. PIEZO channels are thought to sense mechanical forces through flexible transmembrane blade-like domains. Structural studies indeed show that these mechanosensory domains adopt a curved conformation in a resting membrane but become flattened in a membrane under tension, promoting an open state. Yet, recent studies suggest the intriguing possibility that distinct mechanical stimuli activate PIEZO channels through discrete molecular rearrangements of these domains. In addition, biological signals downstream of PIEZO channel activation vary as a function of the mechanical stimulus and of the cellular context. These unique features could explain how PIEZOs confer cells the ability to differentially interpret a complex landscape of mechanical cues.

## Molecular basis of rapid mechanotransduction

In the 1950s, Sir Bernard Katz showed that subjecting a frog muscle to transient stretch causes afferent neurons terminating in the muscle’s spindle to fire action potentials at a rate proportional to the stretch intensity [1]. This observation led Katz to conclude that these neurons must possess a “piezo-electric substance,” capable of converting mechanical force into membrane depolarization. It took scientists many decades following this seminal work to identify the first biological piezo-electric molecule, the bacterial mechanosensitive ion channel of large conductance (MscL) cloned by Sukharev and colleagues [2].

Mechanosensitive ion channels (MSCs) allow ions and other aqueous solutes to diffuse across cell membranes down their chemical or electrochemical gradients in response to the presence of certain mechanical stimuli. MSCs sense mechanical forces transmitted to the channel protein either through lipid–protein interactions (force-from-lipids) or through interactions between the channel and cytoskeletal or extracellular filaments attached to the membrane or its vicinity (force-from-filaments) [3]. Mechanical stimuli transmitted through either pathway are thought to regulate channel activity through force-induced conformational changes, enabling the stimulus to favor certain functional states of the channel protein, the most common ones being defined as closed, open, and inactivated. Stimulus-induced conformational changes occur remarkably fast, often within milliseconds, allowing a nearly instant transduction of mechanical cues into downstream electrochemical signals.

Rapid mechanotransduction provides protection against mechanical stress that could lead to cellular and/or tissue damage. For instance, the transduction of osmotically induced membrane stretch (cell swelling) into osmolyte efflux by MSCs trigger contributes to the maintenance of cell volume through regulatory volume decrease (RVD) responses [4]. In single-cell organisms, which experience severe variations of extracellular osmolarities, the RVD response mediated by MSCs is critical to protect against cell lysis upon osmotic swelling [5-7]. In contrast, animal cells do not experience extreme osmotic shocks under normal physiological conditions and possess large membrane reservoirs in the form of caveolae, microvilli, or invaginations, which can be depleted to buffer osmotically induced membrane stretch [8-10]. In these cells, the RVD response mediated by MSCs mainly contributes to the strict maintenance of cell volume and macromolecular crowding required for proper cytoplasmic and cellular functions such as enzyme activity, fluid transport, and electrical excitability [11-13]. Other classical examples of rapid mechanotransduction are proprioceptive reflexes initiated in the skeletal muscle spindle and Golgi tendon organ of vertebrate organisms. The rapid stretch of the former triggers contraction of the muscle (and inhibition of antagonist’s muscle) to prevent injury of overstretched muscle fibers through the myotatic reflex, whereas rapid stretch of the latter triggers muscle relaxation through the inverse myotatic reflex to prevent damage of muscle and connective tissues under excessive tension.

Today, a dozen of evolutionary-distinct families of genes coding for MSCs have been identified [14]. This suggests that MSCs have emerged independently several times during the course of biological evolution to sense mechanical forces of different nature and/or for different biological purposes. The idea that organisms employ distinct MSCs aligns well with the notion that biomechanical forces encompass a heterogeneous group of biologically discernable stimuli. For instance, the amplitude of biomechanical forces varies over many orders of magnitude from piconewton forces produced by molecular motors [15] to hundreds of kilonewton forces estimated for the bite of *Tyrannosaurus rex* [16]. Biomechanical forces also vary in the direction with which they are being applied with respect to the cell or tissue. For instance, the flow of blood creates tensile forces pushing perpendicularly to blood vessels and also frictional forces (shear stress) exerted parallel to the surface.

## Functional diversity of PIEZO channels

In 2010, the Patapoutian group cloned two homologous membrane proteins, called PIEZO1 and PIEZO2, necessary to confer murine neuroblastoma-derived (N2A) cells and dorsal root ganglia neurons the ability to elicit rapid and transient inward currents in response to a cellular indentation of the cell with a blunt glass probe (commonly referred to as a poking stimulus) [17]. Genes encoding PIEZO proteins have only been identified in eukaryotic genomes, and PIEZO1 and PIEZO2 are the only PIEZO proteins expressed by vertebrates. While PIEZO1 is expressed in most cells and tissues, PIEZO2 is predominantly expressed in peripheral sensory neurons [17]. Interestingly, a discrete number of cells co-express PIEZO1 and PIEZO2. These include a sub-population of baroreceptor endings in the aortic arch and carotid bodies [18], the Meissner mechanoreceptors located in the skin [19], and chondrocytes [20]. In addition, both channels co-localize in the centrosome of many cell lines including C2C12 myoblasts, neuroblastoma-derived N2A cells, and IMCD3 kidney epithelial cells [21].

Follow-up studies have demonstrated that PIEZOs create a cation-selective transmembrane pore that opens in response to mechanical deformations of the lipid bilayer in the absence of other cellular components, demonstrating that PIEZO channels obey the so-called force-from-lipid paradigm [22-24]. Yet, recent studies show that disruption of the cytoskeleton impairs mechanically activated currents mediated by both PIEZO1 and PIEZO2, showing that PIEZO may also obey a force-from-filament paradigm in addition to sensing force from the lipids [25-27].

PIEZO1 is essential for monitoring fluid shear stress in lymphatic capillaries [28,29], in blood vessels [30,31], and at the blood–brain barrier interface [32]. Mice in which the *PIEZO1* gene is invalidated ubiquitously (global *PIEZO1^-/-^*) die before birth but only after the heart starts beating due to disorganized blood vessels, likely because of the lack of shear stress sensing impaired normal vascular development [33]. PIEZO1 senses cell-generated traction forces at focal adhesions, controlling cellular programs such as migration, proliferation, and differentiation [34-39]. Gain-of-function PIEZO1 mutations are linked with red blood cell (RBC) dehydration (also known as xerocytosis or dehydrated hereditary stomatocytosis) [40-43], highlighting an important role of PIEZO1 in mediating RVD in RBCs [44]. PIEZO1 present in RBCs also activates when these cells enter narrow vascular capillaries [45]. Yet, it remains unclear whether PIEZO1 activates when RBCs enter the interendothelial slits of the spleen during splenic filtration [46].

The physiological roles of PIEZO2 have mainly been explored in peripheral sensory neurons. This channel acts as the principal mechanotransducer in proprioceptive neurons innervating muscle spindles and Golgi tendon organ [47,48], revealing that PIEZO2 constitutes Katz’s “piezo-electric substance” speculated while conducting his experiments in the frog muscle [1]. Beyond proprioception, PIEZO2 also acts as an important mechanosensor to transduce gentle touch [49-51] and interoception within many organs and tissues including vascular baroreceptors [18], lungs [52], bladder [53], and the gastrointestinal tract [54]. PIEZO2 activity is strongly potentiated during inflammation [55-57] and is associated with inflammation-related pain states such as allodynia [58,59].

Many studies suggest that the downstream effects of PIEZO channel activation strongly depend on the physiological or cellular contexts. For instance, PIEZO1 activation in stretched epithelial cells promotes cellular division, but PIEZO1 activation in crowded epithelia, wherein cells are compressed as opposed to being stretched, promotes cellular extrusion [60,61]. In addition, PIEZO1 channels often co-localize with calcium-sensitive ion channels, potentially amplifying and diversifying the nature of signaling pathways downstream of PIEZO1 activation [62-67]. Indeed, PIEZO1 colocalizes with BK1 channels in arteries, enhancing their activity in a calcium-dependent manner [62,63]. Furthermore, PIEZO-mediated calcium signals activate various signaling proteins, such as calmodulin, calmodulin-dependent kinases, and Rho GTPases, thereby regulating essential physiological processes including gene expression, cell proliferation, and cytoskeletal remodeling [68,69]. Moreover, the activity of transcriptional co-activators YAP/TAZ and actin cytoskeleton dynamics are also influenced by PIEZO-mediated calcium signaling [70,71].

The intricate signaling network orchestrated by PIEZO channels plays crucial roles in a wide array of physiological and pathophysiological functions, including CaMKII-dependent bone formation [72], CaMKII-Mst1/2-Rac host response during bacterial infection [73], and Rho GTPase linked to cancer metastasis [69]. This diverse range of diseases linked to PIEZO channels highlights their importance in maintaining cellular and organismal homeostasis. By differentially activating downstream molecules with varying calcium sensitivities, PIEZO channels enable cells to fine-tune their responses to distinct mechanical stimuli, ensuring appropriate adaptation to a wide range of physiological contexts.

### How do PIEZOs sense different mechanical stimuli?

MSCs represent a heterogeneous group of evolutionary-unrelated membrane proteins exhibiting totally different primary amino acid sequences, membrane topology, tridimensional structures, and subunit stoichiometries [14]. It is hence anticipated that MSCs exhibit different sensitivities to mechanical stimuli. For instance, the bacterial MscL channel opens in response to hypotonic shocks, gigaseal pressurization, and hydrostatic pressure, but no MscL responses to fluid shear stress or compressional forces have yet been reported [2,74,75]. The epithelial sodium channel ENaC senses fluid shear stress [76,77] and hydrostatic pressure [78] but is not known to detect membrane stretching or osmotic swelling [79]. Members of the OSCA/TMEM63 (hyperosmolarity-gated calcium channels/transmembrane proteins 63) MSC family are sensitive to membrane stretch but differ in their sensitivity to mechanical poking [80,81].

In contrast, PIEZO channels respond to all these types of perturbations in different mechanical assays [17,23,30,31,82-85] as well as less common ones including pillar nano-deflection [86], magnetic pulling [87], and ultrasound [67,88]. The diversity of cell-based mechanical stimulation assays that are effective in activating PIEZOs mirrors the diversity of PIEZOs’ physiological functions. The ability of PIEZOs to sense a wide range of mechanical forces makes them ideal multimodal mechanosensors, capable of transducing a wide range of biomechanical forces experienced by eukaryotic organisms.

**Figure 1:**
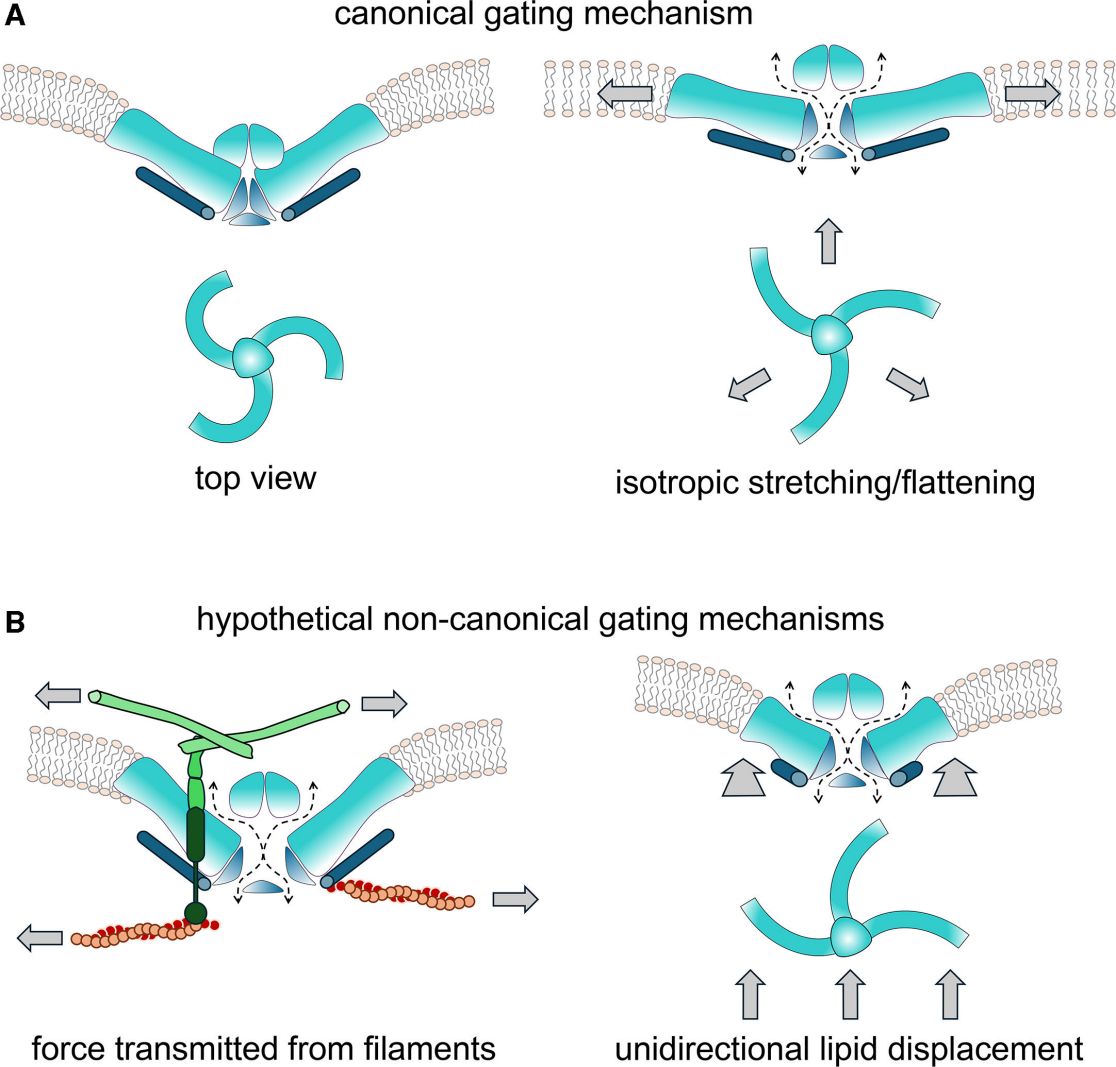
Known and hypothetical force-sensing mechanisms in PIEZO channels. **(A)** Canonical gating mechanism in which membrane stretch/flattening causes the curved PIEZO blades to flatten and open the fenestrated channel pore. **(B)** Hypothetical non-canonical gating mechanisms in which other mechanical stimuli cause distinct conformational changes to open the channel pore, either through a force-from-filament (left) or a force-from-lipid (right) paradigm.

How do PIEZO channels sense membrane tension? PIEZOs consist of the assembly of three large identical subunits, each encompassing ~2500 residues. The homotrimeric complex, which contains 114 transmembrane helices, displays a unique bowl-like architecture with a central ion conduction pore surrounded by three curved peripheral mechanosensory domains called blades [89-95]. Elastic continuous theory predicts that, within a flat membrane, the curved blades compel the membrane to curve around the channel, creating a large footprint of the bent membrane. In turn, due to the non-zero membrane bending rigidity, the curved membrane is predicted to bring about work to flatten the blades [96]. According to this gating mechanism, the amount of work brought about by the membrane footprint to flatten the blades would increase as membrane tension increases, implying that the channel structure would flatten during membrane stretch [97,98]. This canonical curvature-based gating mechanism is consistent with cryo-EM images, showing that the local membrane curvature controls the degree of curvature/flatness of the blades [92,94]. Molecular dynamics simulations of PIEZO1 further revealed that membrane flattening correlates with a flattening of the blades and opening of the pore [99], in agreement with elastic theory and experimental structures (Figure 1a). Beyond modulating channel curvature, membrane tension and curvature also influence the diffusion and localization of PIEZO1 channels in the membrane [100].

How do PIEZO channels respond to other mechanical stimuli? One possibility is that distinct mechanical stimuli, i.e. stimuli delivered through different *in vitro* laboratory apparatus or induced *in vivo* upon distinct physiological processes, converge to produce a common mechanical cue, which is then sensed by the channel’s mechanosensory machinery. The plausibility of this scenario is supported by the notion that PIEZOs tend to be more sensitive to membrane tension than any other known MSC [94,98,101], suggesting that a slight increase in membrane tension caused by any type of mechanical perturbations of the cell membrane could activate the channel.

Another possibility is that PIEZO channels harbor discrete mechanotransduction pathways dedicated to sensing specific types of mechanical deformations of the cell membrane and/or associated cellular structures (e.g. tension, compression, friction, and torque). We find this scenario appealing for several reasons. First, it is now well established that the kinetics of ionic currents mediated by PIEZO channels depend on the nature of the mechanical stimulus. For instance, poke stimuli elicit fast-inactivating currents in both PIEZO1 and PIEZO2, while stretch stimuli applied for an equivalent duration by the patch pipette pressurization method elicit slow-inactivating (PIEZO1) or nearly non-inactivating (PIEZO2) currents [17,102]. Although these differences may be contributed by differential adaptation of the cell to these stimuli [103], this strikingly different kinetics behavior hints that these stimuli could activate these channels through distinct functional pathways.

Second, independent labs have discovered PIEZO mutations that abrogate or strongly reduce the sensitivity of the channel to a specific mechanical stimulus with no or minimal alteration of sensitivity to others [26,90]. For instance, deleting PIEZO2’s fifth intrinsically disordered intracellular loop abolishes PIEZO2-mediated neurite outgrowth and reduces ionic currents evoked by poking stimuli but does not affect stretch-sensitive currents [26]. Similarly, deletion of PIEZO1’s extracellular loops connecting transmembrane helices 15–16 and 19–20 fully eliminates the ability of the channel to produce stretch-sensitive ionic currents but only reduces the amplitude of poking-induced ionic currents [90]. Such a striking stimulus-specificity for a mutant phenotype is difficult to reconcile with the idea that the channel opens and closes its ion permeation pathway through the same molecular mechanism under different stimulation scenarios. These observations make more sense if one assumes that the channel engages distinct conformational pathways to regulate the opening of the pore.

Third, PIEZO is associated with cytosolic regulatory proteins and transmembrane scaffolding proteins, including platelet endothelial cell adhesion molecule-1 (PECAM1), vascular endothelial cadherin (VE-cadherin), integrins, and the cadherin-β-catenin complex [25,104-111]. These complex networks of protein–protein interactions are well poised to facilitate the transmission of mechanical forces through intracellular and extracellular filaments, hence influencing functional states populated in the presence of specific mechanical stimuli. How mechanical forces locally applied to the channel structure through these interactions would bias conformational states remain, however, currently unclear (left panel in Figure 1b).

Fourth, we have recently shown that gentle shear stress activates PIEZO1 through a unique conformational rearrangement of its blades [112,113]. To this aim, we used genetically encoded conformational probes derived from a cyclic permuted green fluorescent protein. We discovered that two independent probes (one inserted at the peripheral tip of the blade and the other within an intracellular disordered loop closer to the pore) elicit large fluorescence signals in response to low-intensity fluid flow stimuli but not to mechanical poking nor osmotic shocks, despite the effectiveness of all tested stimuli to open the channel. What kind of conformational changes might be induced by gentle fluid flow to activate PIEZO1? Although the answer to this question still eludes us, we speculate that this stimulus might promote a unidirectional displacement of lipids in the outer leaflet of the cell membrane, causing asymmetric conformational changes of the channel blades to open the pore (right panel in Figure 1b). An interesting prediction from this hypothetical asymmetric gating mechanism is that some adjacent blades get closer to each other. In contrast, the canonical symmetrical gating mechanism predicts that all blades get physically separated from each other. Future studies will be needed to address this question.

**Perspectives**

- PIEZO channels act as essential mechanotransducers in cells experiencing mechanical stress of different amplitudes and directions.
- Studies combining electrophysiology with mutagenesis or fluorimetry suggest that PIEZOs' unique sensitivity to different mechanical stimuli could be conferred by the presence of distinct mechanotransduction pathways housed within the channel’s mechanosensory domains.
- Structural evidence supporting multimodal gating in PIEZO channels – the existence of distinct conformational pathways by which the channel pore opens in response to distinct mechanical stimuli – is still lacking. Future studies will be needed to answer this important question.

## Abbreviations

MSC: mechanosensitive ion channel
MscL: mechanosensitive ion channel of large conductance
PECAM1: platelet endothelial cell adhesion molecule-1
RBC: red blood cell
RVD: regulatory volume decrease
VE-cadherin: vascular endothelial cadherin
